# The benefits of family planning (FP) use in Benin: an application of the Demographic Dividend Model (DemDiv)

**DOI:** 10.12688/gatesopenres.12906.1

**Published:** 2019-04-02

**Authors:** Justin Dansou

**Affiliations:** 1 Pan African University Institute for Life and Earth Sciences, University of Ibadan, Ibadan, Nigeria

**Keywords:** Family Planning, Benin republic, Demographic dividend, DemDiv, maternal health, child survival

## Abstract

**Background**: Despite the increasing interest in improving access to and utilization of family planning (FP) methods, contraceptives prevalence rates remain low in Benin, and its benefits are not well studied. This study projected FP’s benefits for maternal health and child survival over the Sustainable Development Goals period.

**Methods**: The Demographic Dividend Model created by the Health Policy Project using a large range of data was applied based exclusively on assumptions on FP policy options between 2015 and 2030.

**Results**: It was found that, under the base scenario with no improvements in FP, education and economic variables, however unrealistic, the number of children per Beninese woman would be the same in 2030 as it was in 2015 – about 4.5 children per woman. Benin’s age structure would remain very young and be dominated by dependents. But, FP–scenarios of improvements in contraceptive variables alone showed a negative and linear relationship between FP use and maternal and child deaths. Under the optimistic scenario, increasing access to and use of FP alone from 7.9% (2015) to 33.95% (2030) will save lives of about 200,000 under five year children and 10,000 mothers by 2030. In addition, the average number of children per woman will fall to 3.5 increasing female life expectancy by 5 years. Benin’s age structure will be balanced with more working age people. The country will also record an increase in its human development indicator.

**Conclusion**: To accelerate progresses towards improvement of maternal health and child survival, and get on track in meeting related health targets of SDGs, the present study revealed the importance of strengthening actions toward access to and use of FP in Benin Republic. There is also a need to strengthen education and economic policies to successfully harness the demographic dividend.

## Background

Family planning use has been recognized as a tool for population growth control and a working tool allowing women to control their own reproduction and become more economically active. This is especially relevant in sub-Saharan Africa where population growth, the number of births per woman, and maternal and infant mortality are higher than elsewhere around the globe. In Benin despite recent progress, high maternal mortality persists. With an overall decrease of 29.7% in 15 years, maternal mortality ratio has decreased from 576 to 405 maternal deaths per 100,000 live births over the Millennium Development Goals (MDG) time (between 1990 and 2015) ( WHO *et al*., 2015) in Benin. Early age mortality risks are also challenging. The rate of drop in under 5 years’ age mortality was 44 % (annual rate of reduction 2.4%) from 180 deaths per 1,000 live births in 1990 to 100 deaths per 1,000 live births in 2015. High neonatal mortality persists. Globally, the neonatal mortality rate fell from 36 deaths per 1,000 live births in 1990, to 19 in 2015 and was higher in Benin. With an overall drop of 30.4%, neonatal mortality fell from 46 deaths per 1,000 live births in 1990 to 32 in 2015. The number of under five years’ deaths in Benin was 37,000 in 2015, compared to 39,000 in 1990. Neonatal mortality alone accounts for about one third of all deaths below the age of five years. In 2015, 12,000 neonatal deaths were recorded, while in 1990 10,000 were recorded ( UNICEF, 2015). According to Benin’s census conducted in 2013, half of Beninese aged 6 years or above were illiterate (literacy rate of 6 years or above was 50.6%). Meanwhile, primary schooling rate (6 – 11 years, net) was 56.9 %, and secondary schooling rate (12 – 19 years, net) was 37.4 % ( INSAE, 2016).

Over the past decades, maternal health and child survival have been improving steadily, while the number of children per women remains high in Benin. The current population growth rate between the last two census (2002 and 2013) was 3.5 % (4.8 % in urban area and 2.6 % for rural area). As a result, Benin has a high child dependency ratio resulting in the population be projected to still be growing till the end of the century. The population increased from 2,419,644 in 1960 ( World Bank, 2013) to 10,008,749 in 2013 ( INSAE, 2015), and according to UN projections, will grow to 22,549,000 by 2050, and 35,544,000 by 2100 ( United Nations, 2015). Due to the country’s high fertility, Benin has a large young population in comparison to other countries in sub-Saharan Africa, with more than half (54 %) of its population under the age 20. Benin’s age structure remains very young, dominated by dependents (young dependency ratio – 0–14 years: 46,7 % and old dependency ratio – 65 years and above: 2,7 %) ( INSAE, 2016). The number of births per woman fell from 7 in 1980 to about 5 (4.8) in 2013. The lower level of contraceptive prevalence rate accounts the higher fertility rate. Benin’s demographic and health surveys (BDHS) conducted in 2001, 2006 and 2011/2012 reported the following rates of modern contraceptive prevalence among women in union 6.8%, 6.7% and 8.8% respectively. Meanwhile, traditional contraceptives uptake has decreased, falling from 10.8% in 2001, to 9.8% in 2006, and 5.0% in 2011/2012. However, knowledge of contraceptive methods is relatively high in Benin. Most women (85%) investigated during the 2011/2012 DHS knew at least one contraceptive method ( INSAE & ICF, 2013).

Recently, the benefits of family planning use have become a core component of the new concept, demographic dividend, developed to highlight supports that changes in age structure of a population could provide for the economic development of that country. The concept has, then, become a key component drawing attention of policy makers where potential economic benefits of the demographic dividend and the policies required to achieve it are well-documented at the global level. An empirically sound projection model that can be readily applied in any high- fertility country to project demographic dividend ( Moreland *et al*., 2014) is now available.

Applied in Kenya’s context, the model reported that increasing access to and use of family planning will save the lives of more than 5 million children and 350,000 mothers, and balance Kenya’s age structure, with the working – age population growing to 73 percent of the total population in 2050 ( NCPD & HPP, 2014). In Uganda, changes in family planning use was among the three leading scenarios providing the country the maximum advantage for demographic dividend harnessing. To maximise the demographic dividend (socioeconomic transformation envisaged in vision 2040), investments in family planning and female education to reduce fertility and the high child-dependency burden were revealed among the key areas ( Uganda National Planning Authority, 2014). In Cameroon, the advantages in meeting family planning needs among women are numerous; first, the current use of contraceptive (in 2014) decreased the worst outcomes of pregnancy and childbirth related complications (avoided unwanted pregnancies representing 900 maternal deaths averted) to about 11 % to 17 % relative to a situation of no use of contraceptive. Additionally, if the current needs in family planning among Cameroonian females are met at 50%, unplanned pregnancies will be reduced by 38 % (about 187,000 unplanned pregnancies averted) each year, 95,000 unplanned births will be averted, as well as 65,000 induced abortions and 600 maternal deaths ( Vlassoff *et al*., 2014).

Although the Demographic Dividend Model ( Health Policy Project and USAID, 2014) provides means to evaluate the impacts of policies from different sectors including FP, education and economic), the focus of this study is single sector policies. Single sector policies contribute to development and are measurable. Moreover, even though the demographic dividend model used is conceived for multisector impact evaluation, it also allows to examine the impact of single sector policies. This study applied the DemDiv ( Health Policy Project and USAID, 2014), to explore the impact of family planning policy options in Benin for maternal health and child survival over the Sustainable Development Goals time (2015~2030). More specifically, it explored maternal and child deaths that could be averted between 2015 and 2030 based on three family planning policy options.

## Methods

### Overview of DemDiv model

The demographic dividend is a temporary opportunity for faster economic growth that begins when fertility rates fall, leading to a larger proportion of working-age adults and fewer young dependents ( Gribble & Bremner, 2012; NCPD & HPP, 2014). Context-specific investments in family planning, education, and the economy are the key areas targeted to harness the demographic dividend, a complex concept. The Demographic Dividend Model ( Health Policy Project and USAID, 2014) used was created by the Health Policy Project (HPP), with support from United Stated Agency for International Development (USAID) ( Moreland *et al*., 2014). It addresses the complexity of the demographic dividend by linking age structure with social and economic development, enabling policymakers to quantify the changes that would be required to successfully achieve a demographic dividend. In other words, DemDiv projects the impacts of policies from three key areas (FP, education and economic) under scenarios. The model is designed to show how the combined power of multisectoral policy investments in FP, education and economy can generate a dividend not possible under the status quo. It also gives the possibility to evaluate the impact of investments in each area independently. Overall, DemDiv is developed in a two-part model describing demographic and economic changes. The demographic component underlies the model structure, projecting child mortality, dependency ratio, fertility, population size and structure, and life expectancy. Demographic calculations then feed into the economic model, which consists of three equations describing capital formation, employment growth, and total factor productivity as a function of age structure and other social and economic variables ( Moreland *et al*., 2014).

### Study assumptions

To assess the impacts of family planning use, two scenarios were developed for the period 2010 – 2050. Though the period covered 2010 – 2050 in order to meet one of the basic requirements of the model, the focus of the current study was 2015 – 2030. The two scenarios made were:

1) First scenario: a base case scenario of no changes in FP, education and economic policies variables. With this scenario, all education, family planning and economic policies variables remained unchanged as they were in 2010 ( Table 1) over the projection time,
2) Second scenario: an FP – only scenario of improvements in contraceptive variables alone. Only family planning policy variables changed over time till the end year of projection while all other variables ( Table 1) kept constant. Prospects on changes in FP use were made based on recent changes in FP use through previous commitments and on plan of actions for future FP projects. Changes in FP use according to BDHS conducted in 2001 and 2011/2012 showed an average annual increase in modern contraceptive prevalence rate of 2.07% (mCPR increased from 6.8% in 2001 to 8.8% in 2011/2012) while traditional contraceptive prevalence rate has dropped with an annually rate of about 10.55% (from 10.8% in 2001 to 5.0% in 2011/2012). The government’s Family Planning 2020 (FP2020) commitment is to increase modern contraceptive use from 8.7% in 2013 to 20% by 2018. Based on this optimistic commitment, modern contraceptive prevalence rate will reach the highest level of 100% well before 2050 if current FP commitments would continue beyond 2018. This is however, very ambitious. The present study envisaged an increase in contraceptive use according to three hypotheses:
  ○ Low hypothesis: modern contraceptive use will increase following the previous rate of increase (2.07% annually) to reach the level of 17.9% by 2050;
  ○ Moderate hypothesis: this is the medium of the low hypothesis and the most optimistic hypothesis. Modern contraceptive use will, then, increase to reach the level of 38.9% by 2050;
  ○ Most optimistic hypothesis: this is based on current family planning 2020 commitments. Modern contraceptive use will reach the top level of 60% by 2050.

Traditional contraceptive prevalence rate (already low) is assumed to remain the same under each of the three hypothesises. It will decrease from 5% to 2% between 2010 to 2050.

**Table 1.** Education, family planning and economic policies base variables for the DemDiv for the base year (2010).

SECTOR Variable	Value	Data source
**EDUCATION**		
Expected years (Female)	9.42	UNESCO UIS Database Latest Data Available 2005–2012
Expected years (Male)	12.68	
Mean years (Female)	2.06	Barro & Lee Database 2014, 2010 Data
Mean years (Male)	4.55	
Mean years (Both)	3.31	
**FAMILY PLANNING**		
CPR modern (Married Women)	7.9	Demographic and Health Survey, STAT compiler Latest Data Available 2005–2013
CPR traditional (Married Women)	5.0	
Postpartum Insusceptibility (Months)	12.3	
Sterility (Percent All Women 45–49)	4.1	
**ECONOMIC POLICIES**		
GCI 1A: Public Institutions	2.95	World Economic Forum, Global Competitiveness Report 2013–2014
GCI 6.14: Imports as a %GDP	38.10	
GCI 7A: Labor Market Flexibility	3.39	
GCI 8A: Financial Market Efficiency	2.96	
GCI 9B: ICT Use	3.20	

UNESCO UIS: United Nations Educational, Scientific and Cultural Organization Institute for Statistics, CPR: contraceptive prevalence rate, GCI: Global Competitiveness Index *Source: DemDiv base values*

### Data

The model contains default data from international and official sources, but also allows users to customize it by entering context-specific data and set all future policy goals. This study relied on data provided by the model ( Health Policy Project and USAID, 2014). Missing data and gaps were filled. The period of projection provided by model was 2010 – 2050, but the focus of the current study was the Sustainable Development Goals time – 2015 – 2030. Therefore, the base year for the projection was set at 2010. Corresponding data for the base year, however, ranged between 2011 – 2013.

Table 1 presents the base variables for education, family planning and economic policies used by the DemDiv. At the level of education, related policy variables include expected years of education (female 9.4 years and male 12.7 years) and mean years of education. Family planning variables include modern (7.9%) and traditional (5.0%) contraceptive prevalence rates among married women, postpartum insusceptibility (12.3 months) and sterility level among all women aged 45 – 49 years (4.1).

Table 2 shows the expected value for family planning policy variables by the end of SDGs time (2030) drawn from estimates based on the end values set for the model (2050). By 2030, modern contraceptive rate among married women is expected to reach the level of 33.95% under the optimistic scenario while traditional contraceptive prevalence rate will drop to 3.25%, postpartum insusceptibility will remain 12.3 months, while female sterility between 45 – 49 years will fall to 3% (see Underlying data ( Dansou, 2019)).

**Table 2.** Family planning policy variables set for the end year of the projection (2030).

Indicator	Base scenario	Low hypothesis	Moderate hypothesis	Optimistic hypothesis
CPR modern (married women)	7.9%	12.90%	23,43%	33.95%
CPR traditional (married women)	5.0%	3.50%	3.50%	3.50%
Postpartum Insusceptibility (months)	12.30	12.30	12.30	12.30
Sterility (percent all women 45–49)	4.1	3.55%	3.55	3.55

CPR: contraceptive prevalence rate *Source: Based on the country’s vision and the Sustainable Development Goals targets*.

In addition to policy variables, 19 additional baseline values pertaining to health and economy were used. They were: percent married/in union women (70.4%), total fertility rate (4.9), percent births at any risk (52.3%), infant mortality rate (46), under-five morality rate (75), maternal mortality ratio (340), contraceptive effectiveness (0.95 for modern and 0.5 for traditional method), female life expectancy (59.6 years), capital formation per capita (141), initial employment (ages 15 and older: 4 133 182), employment growth rate (3.4%), Gross Domestic Product (GDP) per capita (2010 constant $US: 605), GDP growth rate (3.7%), capital stock growth rate (0.5%), female-male life expectancy difference (2.74 years), capital stock depreciation rate (4%), primary education costs as a percentage of GDP (15.02%) per capita, labour force participation rate (1) and ratio of capital stock to population aged 15 years and older per capita (11,190). All these variables were the baseline values of the DemDiv except the ratio of capital stock to population 15+ per capita drawn from ( Feenstra *et al*., 2015) and ( INSAE, 2016).

## Results

Even with the focus of family planning sector alone, the modelling exercise provided benefits of family planning use as those underlying more sectors highlighted in other settings ( NCPD & HPP, 2014; Uganda National Planning Authority, 2014; Vlassoff *et al*., 2014). The modelling exercise shows that under the base scenario, however unrealistic, with no improvements in FP, education and economic variables, the fertility rate would remain the same in 2030 as it was 2015 – about 4.5 children per woman. Benin’s age structure would remain very young and be dominated by dependents – dependency ratio estimated at 77%. In addition, Benin’s Gross Domestic Product per capita will remain lower than the level of each of the three FP scenario.

In contrast, the benefits of family planning use for maternal health and child survival vary drastically in proportion of the level of achievement in access to and use of contraceptives – the greater the achievement, the greater the dividends in term of gains in maternal and child deaths averted. Table 3 presents the benefits of family planning use for maternal health according to the three family planning options. Its benefits for under five year children are presented in Table 4, while Table 5 presents the gain for children below the age of one year. Overall, by the end of SDGs time, improvements in family planning uptake alone keeping all other policy variables constant would help avert approximatively between 2,000 and 10,000 maternal deaths according to family planning hypotheses.

**Table 3.** Benefits of family planning (FP) use for maternal health.

Year	Cumulative maternal deaths				Cumulative maternal deaths averted		
	*Base* *scenario*	*Low* *hypothesis*	*Moderate* *hypothesis*	*Optimistic* *hypothesis*	*Low* *hypothesis*	*Moderate* *hypothesis*	*Optimistic* *hypothesis*
2015							
2020	6 954	6 600	5 781	5 052	355	1 174	1 902
2025	14 992	13 985	11 729	9 809	1 007	3 263	5 183
2030	24 161	22 142	17 760	14 196	2 019	6 401	9 965

*Source: DemDiv outputs*

**Table 4.** Benefits of FP use for under five year children survival.

Year	Cumulative under five deaths				Cumulative under five deaths averted		
	*Base* *scenario*	*Low* *hypothesis*	*Moderate* *hypothesis*	*Optimistic* *hypothesis*	*Low* *hypothesis*	*Moderate* *hypothesis*	*Optimistic* *hypothesis*
2015							
2020	141 883	135 656	120 595	106 315	6 227	21 288	35 568
2025	306 058	288 109	245 361	205 800	17 949	60 697	100 258
2030	493 792	457 363	372 029	294 932	36 430	121 763	198 860

*Source: DemDiv outputs*

**Table 5.** Benefits of family planning (FP) use for infant survival.

Year	Cumulative infant deaths				Cumulative infant deaths averted		
	*Base* *scenario*	*Low* *hypothesis*	*Moderate* *hypothesis*	*Optimistic* *hypothesis*	*Low* *hypothesis*	*Moderate* *hypothesis*	*Optimistic* *hypothesis*
2015							
2020	94 088	90 217	80 882	72 065	3 871	13 206	22 022
2025	202 833	191 796	165 570	141 388	11 037	37 263	61 445
2030	326 883	304 654	252 668	205 949	22 230	74 216	120 934

*Source: DemDiv outputs*

According to the hypothesis of the lowest change in family planning based on the previous increases taking modern contraceptive prevalence rate from 7.9% in 2010 to 12.9% by 2030, about two thousand (2,019) maternal deaths will be averted. The moderate hypothesis, conciliating pessimists (low hypothesis) and most optimists (based on current family planning 2020 commitments) projecting modern contraceptives use to reach the level of 23.43% by 2030, a total of 6,401 maternal deaths will be averted while 9,965 mothers will be saved from dying maternal deaths if commitments and actions take modern contraceptives use to the level 33.95% within the same period. Under this assumption, more than two out of every five maternal deaths will then be averted by 2030. Indeed, under the base case scenario of no improvement in FP, education and economic, a total of 24,161 maternal deaths will be recorded between 2015 and 2030, while the achievement of the optimistic hypothesis will record 14,196 maternal deaths ( Table 3). Increasing access to and use of family planning will save the lives of approximatively 200,000 children under 5 years by 2030 (121,763 for the moderate hypothesis and 36,430 for the low one) if actions and commitments meet optimistic expectations ( Table 4). For infant mortality, the gains will be 22,230; 74,216 and 120,934 for low, moderate and optimistic hypothesis respectively ( Table 5).

**Figure 1.**
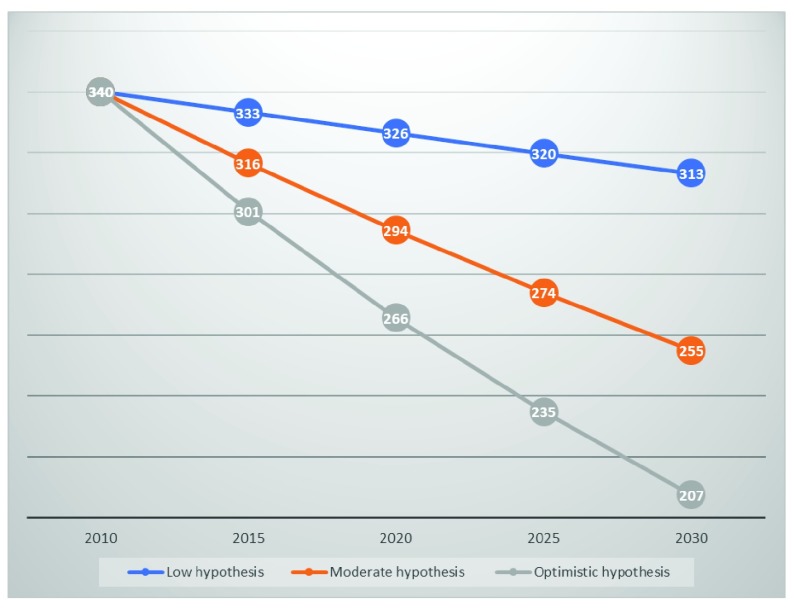
Estimates in maternal mortality ratio. *Source: Data from DemDiv outputs*

The decrease in maternal and under five year children deaths implies a decrease in risks of mortality of both mother and child. A drastic decrease in maternal and child risks of mortality will be then recorded in proportion of the changes in family planning use. Figure 1 shows the estimates in maternal mortality ratio according to the three different family planning scenario. The optimistic scenario of high achievement in modern contraceptives use will reduce the maternal mortality ratio from its current level (340 per 100,000 life births) to 207.5 maternal deaths for every 100,000 life births. The risk of maternal death will then be reduced by 39%. According to the moderate hypothesis, maternal mortality ratio will be expected to drop to the level of 265.6 maternal deaths for 100,000 live births while under the low hypothesis, about 300 mothers out of every 100,000 will still be dying during pregnancy and childbirth ( Figure 1).

**Figure 2.**
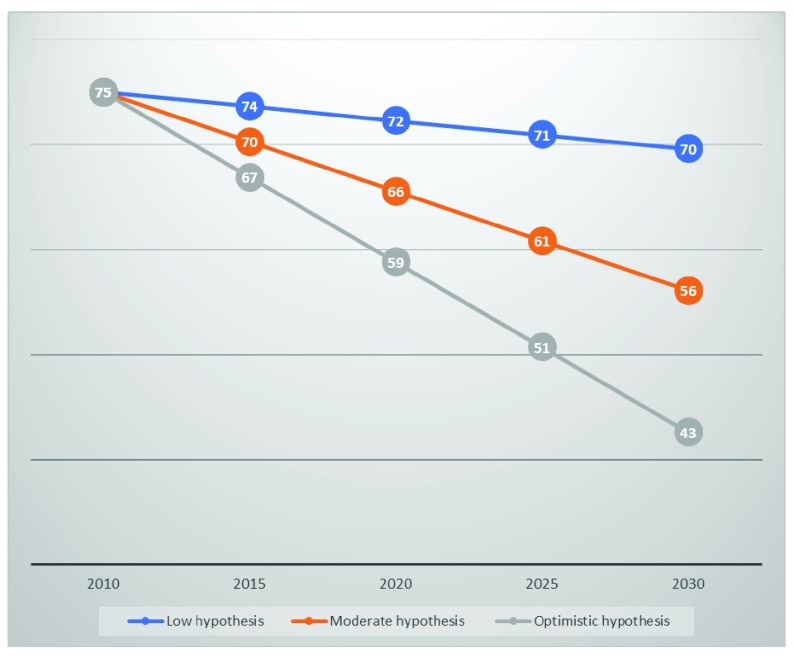
Estimates in under five-year mortality ratio. *Source: Data from DemDiv outputs*.

Like maternal mortality ratio, under five-year mortality rate decreases by increasing family planning uptake. Figure 2 presents the distribution of the estimates of under-five mortality rate between 2015 and 2030 according to the three different family planning options. A high increase in modern contraceptive use will reduce the under-five mortality rate by 42.6 deaths per 1,000 life births relative to its current level, 75 deaths per 1,000 life births (2015). The achievement of the low hypothesis will reduce the under-five mortality risk by 10% by 2030.

In addition of its positive impact for both maternal health and child survival, family planning uptake is also revealed to have numerous others positive impacts for human development in Benin. Among them, the creation of a large work force, which, is well-educated and in good health will enhance the economic productivity of the country. Under the optimistic scenario, Benin will record about three (3.48) children per woman by 2030, where woman will gain about five years more on their current life expectancy (increasing from 61.5 years in 2015 to 66.8 in 2030). As a result, Benin will have its human development indicator increased from 0.37 to 0.40 within the same time.

## Conclusion and policy implications

Family planning is the heart of the demographic dividend concept even if all three components (family planning, education, and economy) of the concept are dependents and requiring work across sectors. High demand for children, low family planning uptake and high maternal and child mortality were among the main challenges that Benin faces in its efforts for socioeconomic development of the country by reducing fertility. The current study examined the benefits of FP use focusing on its benefits for both mothers and children below the age of five years using Demographic Dividend Model (DemDiv) created by the Health Policy Project. To avert thousands of maternal and children deaths and undergo a steady fertility decline to reach an age structure concentrated into the working ages, Benin must strengthen policies toward voluntary family planning programmes enabling couples to realise their reproductive preferences while simultaneously improving education and economic environments. Under an unrealistic scenario of no improvement in education and economic, family planning use alone which was the focus of the current investigation will save lives of thousands of mothers and children and contribute to the economic environment improvement and population well-being. Under the most optimistic scenario based on Benin government’s Family Planning 2020 (FP2020) commitments increasing modern contraceptive rate to just 33.95% by 2030, Benin will save lives of about 10,000 mothers from dying maternal deaths and about 200,000 children from dying preventable deaths before their fifth birthday.

Despite such results, the gains will be much greater when policies reach the two other areas needed to fully harness the demographic dividend - namely investments in human capital and the implementation of sound economic policies and investments in high-quality education as well as vocational training - are needed to harness the dividend that the changes in age structure resulting from fertility decline will provide.

## Data availability

### Source data

Health Policy Project: Demographic Dividend Model (DemDiv). http://www.healthpolicyproject.com/index.cfm?id=software&get=DemDiv ( Health Policy Project and USAID, 2014)

### Underlying data

Open Science Framework: The benefits of family planning (FP) use in Benin: an application of the Demographic Dividend Model (DemDiv). https://doi.org/10.17605/OSF.IO/RC5ZQ ( Dansou, 2019)

This project contains the following underlying data:

- CC0_Licence_489_DemDivModelFINAL_LowHypothesis_DATA_Benin.csv (Low hypothesis data)
- CC0_Licence_489_DemDivModelFINAL_ModerateHypothesis_DATA_Benin.csv (Moderate hypothesis data)
- CC0_Licence_489_DemDivModelFINAL_OptimisticHypothesis_DATA_Benin.csv (Optimistic hypothesis data)

Data are available under the terms of the Creative Commons Zero "No rights reserved" data waiver (CC0 1.0 Public domain dedication).

## References

[ref-1] Dansou J: The benefits of family planning (FP) use in Benin: an application of the Demographic Dividend Model (DemDiv).2019 10.17605/OSF.IO/RC5ZQ PMC6826173 31723727

[ref-2] Feenstra RC Robert I Marcel PT: The Next Generation of the Penn World Table. *Am Econ Rev.* 2015;105(10):3150–3182. 10.3386/w19255

[ref-3] Gribble JN Bremner J: Achieving a demographic dividend. *Popul Bull.* 2012;67(2). Reference Source

[ref-4] Health Policy Project and United States Agency for International Development (USAID): DemDiv Model. Washington, DC: Futures Group, Health Policy Project.2014 Reference Source

[ref-6] INSAE: *RGPH 4: Que retenir des effectifs de population en 2013? * 2015; INSAE, Cotonou. Reference Source

[ref-7] INSAE: *Principaux Indicateurs Sociodémographiques et Economiques (RGPH-4, 2013).*Cotonou, Bénin: INSAE.2016.

[ref-8] INSAE & ICF: *Rapport de l'Enquête Démographique et de Santé du Bénin 2011-2012.*Calverton, Maryland, USA: INSAE et ICF International:2013 Reference Source

[ref-9] Moreland S Madsen EL Kuang B: Modeling the Demographic Dividend: Technical Guide to the DemDiv Model. Washington, DC: Futures Group, Health Policy Project.2014;44 Reference Source

[ref-10] NCPD, HPP: Demographic dividend opportunities for Kenya: Results from the DemDiv model.National Council for Population and Development - NCPD and Health Policy Project - HPP.2014 Reference Source

[ref-11] Uganda National Planning Authority: Harnessing the demographic dividend: Accelerating socioeconomic transformation in Uganda.2014 Reference Source

[ref-12] UNICEF: *Levels & trends in Child mortality: Report 2015, Estimates Developed by the UN Inter-agency Group for Child Mortality Estimation* 2015 Reference Source

[ref-13] United Nations: World Population Prospects 2015, Data booklet. *2015 Revision* 2015 Reference Source

[ref-14] Vlassoff M Jerman J Beninguisse G: Avantages à répondre aux besoins de contraception des Camerounais. *In Brief* New York: Guttmacher Institute.2014;(2) Reference Source

[ref-15] WHO, UNICEF, UNFPA, *et al.*: *Trends in maternal mortality: 1990 to 2015.*Geneva:2015 Reference Source

[ref-16] World Bank: *World Development Indicators* 2013 Reference Source

