# Supplementary material for: The benefits of family planning (FP) use in Benin: an application of the Demographic Dividend Model (DemDiv)
Source: Gates Open Res. 2019 Apr 2;3:1110. [Version 1] doi: 10.12688/gatesopenres.12906.1 (PMC6826173; doi:10.12688/gatesopenres.12906.1)
Supplement: Peer review history

# Peer review: Reviewer response for version 1

**DOI:** 10.21956/gatesopenres.14002.r27691

**Published:** 2019-11-01

**Authors:** James Kiarie

**Affiliations:** 1 Department of Reproductive Health and Research, World Health Organization (WHO), Geneva, Switzerland; 2 Division of Obstetrics and Gynecology, University of Nairobi, Nairobi, Kenya

This article projects the health benefits in 2030 of improving FP use in Benin using the Demographic Dividend Model. The paper explores maternal and child health benefits under four scenarios of change in modern contraceptive prevalence from a 2010 baseline while assuming no change in education and economic policies variables. The scenarios of contraceptive prevalence change are: no change; increase following previous rate of increase (low); increase to meet the FP 2020 commitments (optimistic) and an increase that is the medium of the low and optimist (moderate).

 This modelling study clearly demonstrates the positive impacts of increasing contraceptive prevalence for maternal and child health even without changes in education and economic policies variables. Family planning uptake is also shown to have numerous other positive impacts for human development. Although in discussing the results the authors focus on the optimistic scenario the moderate scenario is probably more likely based on recent surveys.

 In interpreting the study, it is however important to consider the limitations of projections inherent with use of the demographic dividend models particularly failure to include some linkages between population growth and economy. These are likely to be important with climate change and advancements in technology. Many of these limitations and the fact that the authors are assuming no change in education and economic policies variables mean that the benefits of increasing modern contraceptive prevalence in Benin maybe greater than reported.

I confirm that I have read this submission and believe that I have an appropriate level of expertise to confirm that it is of an acceptable scientific standard.

# Peer review: Reviewer response for version 1

**DOI:** 10.21956/gatesopenres.14002.r27686

**Published:** 2019-10-08

**Authors:** Akanni Akinyemi

**Affiliations:** 1 Department of Demography and Social Statistics, Obafemi Awolowo University, Ile Ife, Nigeria

- The manuscript is well written and contributes significantly to the demographic dividends discourses with reference to Benin.
- The article provides further evidence for the benefit of investment in family planning on child survival and improved maternal health. The models and datasets are very adequate. The results section is well focused and apt. 
- The conclusion and policy implications section needs to be linked to the results. The conclusion is too general. What are the specific requirements to achieve improved child survival and also for better maternal health indicators? 

I confirm that I have read this submission and believe that I have an appropriate level of expertise to confirm that it is of an acceptable scientific standard.
